# Aloe-Vera: A Nature’s Gift to Children

**DOI:** 10.5005/jp-journals-10005-1059

**Published:** 2010-08-17

**Authors:** Neha Gupta, Manohar Bhat, Prabha Devi, Girish

**Affiliations:** 1 Postgraduate Student, Department of Pedodontics and Preventive Dentistry, Jaipur Dental College, Jaipur, Rajasthan, India; 2 Professor and Head, Department of Pedodontics and Preventive Dentistry, Jaipur Dental College, Jaipur, Rajasthan, India; 3 Senior Lecturer, Department of Pedodontics and Preventive Dentistry, Jaipur Dental College, Jaipur, Rajasthan, India; 4 Reader, Department of Oral Pathology, Jaipur Dental College, Jaipur, Rajasthan, India

**Keywords:** Aloe-Vera gel, deciduous teeth, pulpotomy, histopathological sections.

## Abstract

^1^Aloe-Barbadensis Mill (Liliaceae) is used in the traditional medicine of Mexico and other countries for anti-inflammatory and cosmetic purposes (Diez-Martinez 1981, Grindlay and Reynolds 1986). Two components are obtained from the fresh leaves of Aloe-Barbadensis, a bitter yellow juice (exudate), which drains from the transversally cut leaves used as a laxative (Ishii et al 1990) and a mucilaginous gel from leaf parenchyma, which has been used as a remedy for a variety of pathological states such as arthritis, gout, acne, dermatitis, burns and peptic ulcers induced by epithelial alterations (Cap-passo and Ganginella 1997, Reynolds and Dweek 1999).

The aim of this study is to evaluate efficacy of Aloe-Vera gel as a healing agent in an endodontic procedure called pulpotomy. Fifteen primary molars were treated for pulpotomy using ‘Aloe-Vera gel’. Patients were recalled after 1 month to check for any clinical symptoms. None of the patients reported with clinical symptoms of pain, mobility, abscess and histopathological evaluation done following extraction after 2 months showed positive signs of healing.

## INTRODUCTION

Maintenance of the integrity of primary dentition until their normal exfoliation is important for proper development and maturation of the child, proper growth of facioskeletal complex to its full potential and for its good occlusion with its good esthetic qualities. Thus, primary teeth with pulpal and periapical problems should be treated by endodontic therapy, which depends on reduction or elimination of bacteria from root canals.^2^ Lots of materials have been tried as a pulp therapy agent in deciduous teeth with each of them having their own advantages and disadvantages.

Any derivative from nature is gods given gift and thus natural products are always a source of attraction of all. Use of such products is increasing in fields of dentistry. One such product which is used in our study is ‘Aloe-Vera’ as an endodontic procedure agent for ‘pulpotomy’ in children.

## METHODS

Twenty-five children who visited OPD of Department of Pedodontics, Jaipur Dental College, were selected for the study. Out of 25 screened patients, 15 were finalized for the study. The selection was done so that children who were healthy were chosen with at least one carious primary molar indicated for pulpotomy.

**Fig. 1:**
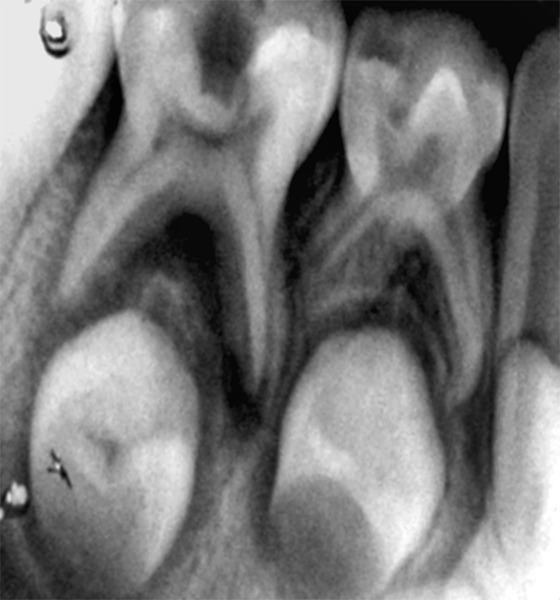
Preoperative radiograph in relation to 74

The following are the inclusion criterias for the study^3^ (Fig. 1).

1. Tooth should be vital with healthy periodontium.
2. Pain if present should neither be spontaneous nor persistent.
3. Tooth should be restorable.
4. Tooth should possess at least 2/3rd of the root length.
5. Hemorrhage from the amputation site should be pale red and easy to control.
6. Children who were to go for serial extraction procedure.
7. Antibiotics should not be received by patient at least one week prior to the treatment.

The following were the exclusion criterias for the study.^3^

1. Evidences of internal resorption.
2. Presence of any interradicular bone loss.
3. Existence of abscess or fistula in relation to the tooth.
4. Radiographic signs of calcific globules seen in pulp chamber.
5. Caries penetrating floor of pulp chamber.
6. Tooth close to natural exfoliation.

**Fig. 2:**
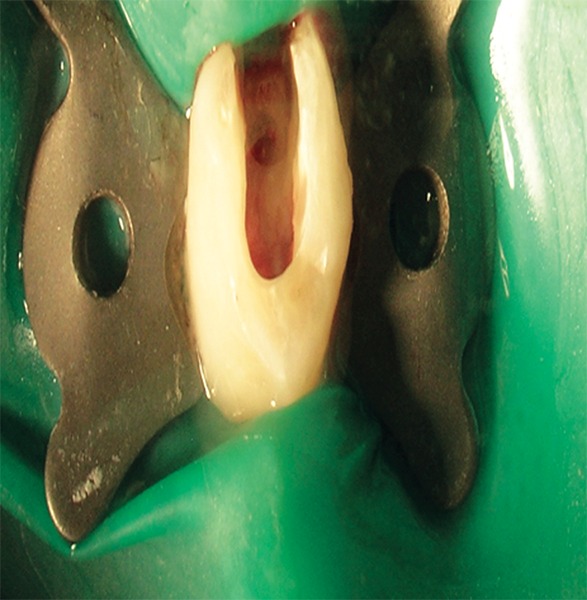
Access cavity opening for pulpotomy in relation to 74

**Fig. 3:**
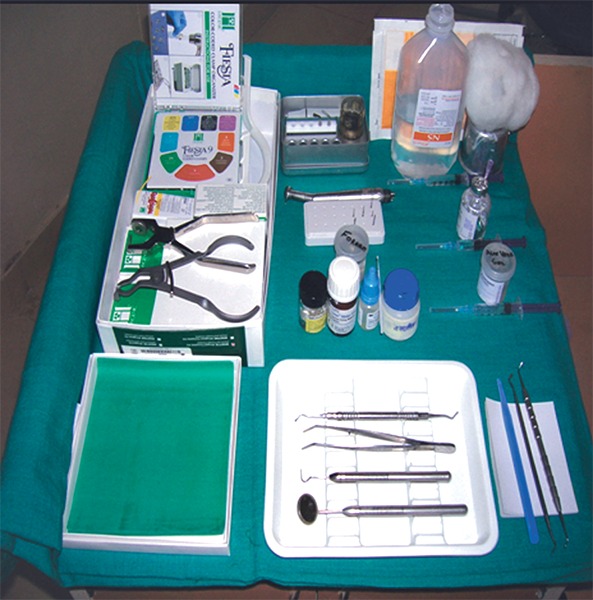
Armamentarium used for pulpotomy

**Fig. 4:**
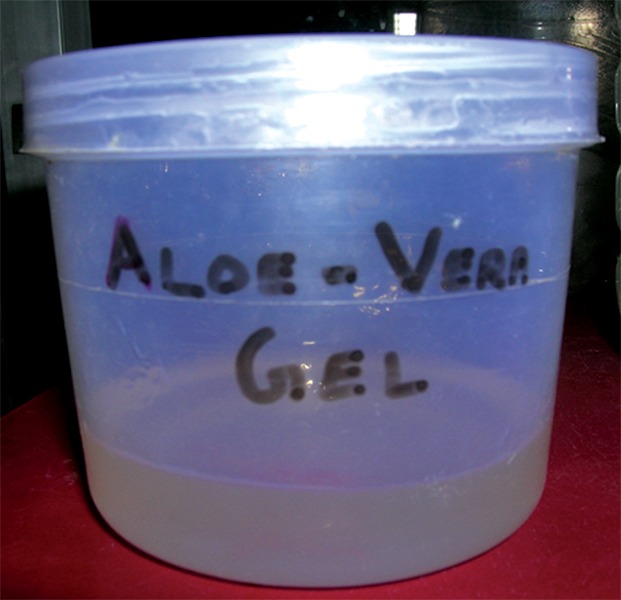
Aloe-Vera gel used for pulpotomy

## PROCEDURE

**Fig. 5:**
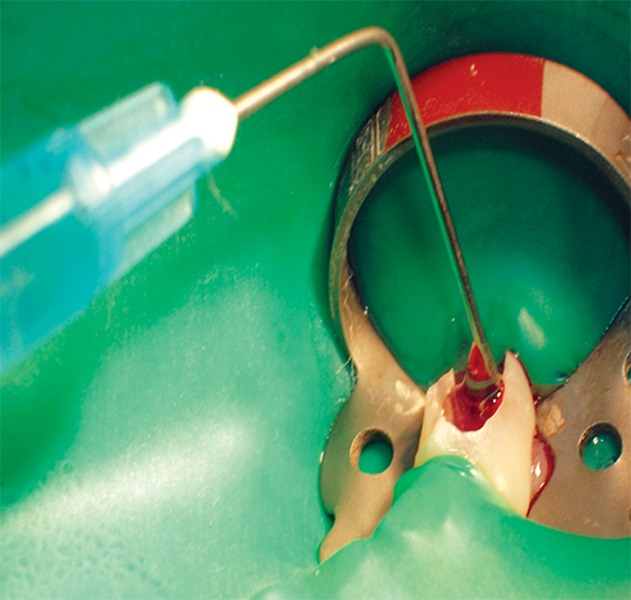
Placement of Aloe-Vera gel over pulp stumps

**Fig. 6:**
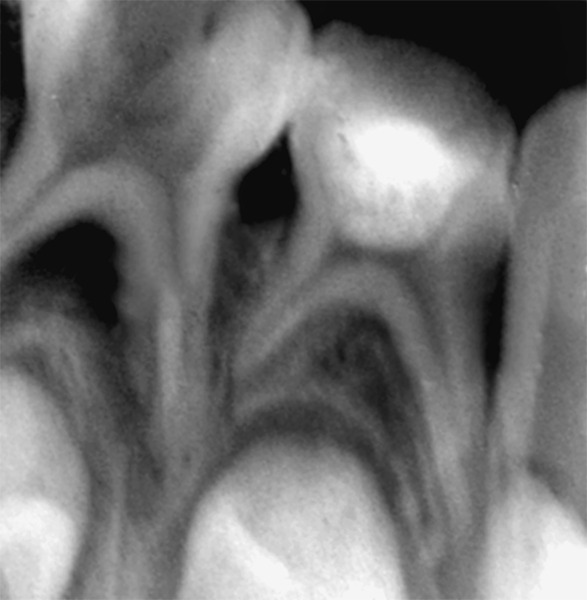
Postoperative radiograph in relation to 74

Under ideal conditions of sterilization and isolation with rubber dam, access cavity opening was done on the tooth selected (Fig. 2). Routine armamentarium which is used during pulpotomy procedure was used along with freshly prepared Aloe-Vera gel (Figs 3 and 4). Then coronal pulp was removed using spoon-excavator and the sample is sent for histopathological examination. The pulp chamber was cleaned properly with normal saline, followed by hemostasis with wet cotton pellets. Then Aloe-Vera gel loaded in syringe was placed over each root stump (Fig. 5). This was then followed by placement of noneugenol containing temporary restorative material provipast and then final restoration of the cavity was done with ketac molar GIC (Fig. 6). Patient were then recalled after 30 days for checking-up any clinical symptoms of pain, mobility, abscess and then finally recalled after 60 days for checking vitality of the teeth.

Extractions of all the teeth were done after taking consent from parents. Each extracted tooth was kept in formalin containing container and sent for histopathological examination to department of oral pathology. Space maintainer if required was given for the teeth which were extracted.

### Preparation of Slides

Decalcification of the tooth samples was done by using acid and the sample was washed in running water for about hours. The sample was then processed by routine tissue processing methods and embedded in paraffin wax block. Sections of 3 to 4 μm thick were obtained and were stained using routine H and E staining procedure. The stained sections were mounted by cover slips using DPX and were observed under light microscopy.

## RESULTS

### Clinical Findings

All the teeth in which pulpotomy was done showed no signs of abscess, mobility, pain after 2 months from the date of completion of procedure.

### Histological Findings

The decalcified sections of the extracted teeth showed intact radicular pulp with features of vitality like delicate fibro-cellular connective tissue stroma, blood vessels, intact odontoblastic layer, few chronic inflammatory cells and extravasated RBC’s.

**Fig. 7:**
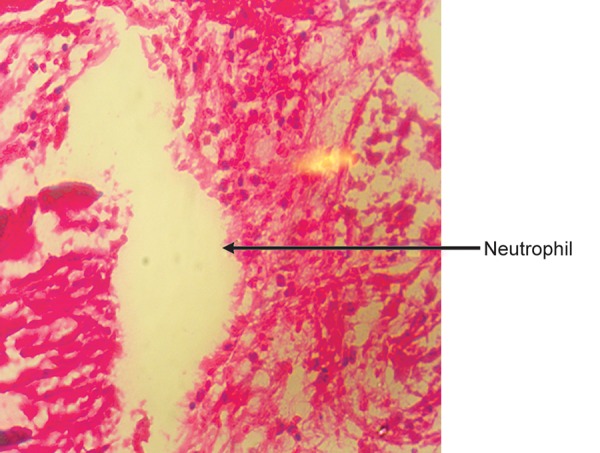
Coronal pulp (H & E staining 10X)

Coronal pulp under 10X magnification showed presence of neutrophils (Fig. 7).

**Fig. 8:**
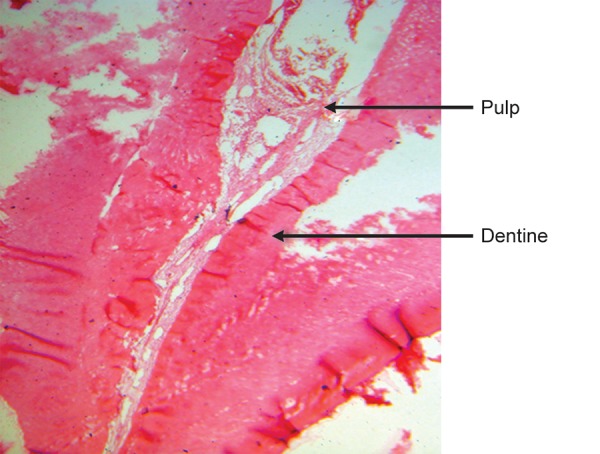
Root canal containing pulp tissue enclosed by root dentine (H & E staining 4X)

Cross-section of root canal under 4X magnification shows pulp tissue enclosed by root dentine (Fig. 8).

**Fig. 9:**
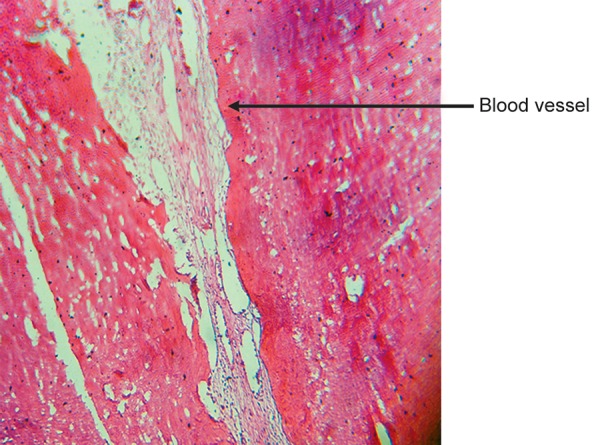
Root canal containing vital pulp tissue enclosed by root dentine (H & E staining 10X)

Cross-section of root canal under 10X magnification shows vital pulp tissue containing blood vessels enclosed by root dentine (Fig. 9).

**Fig. 10:**
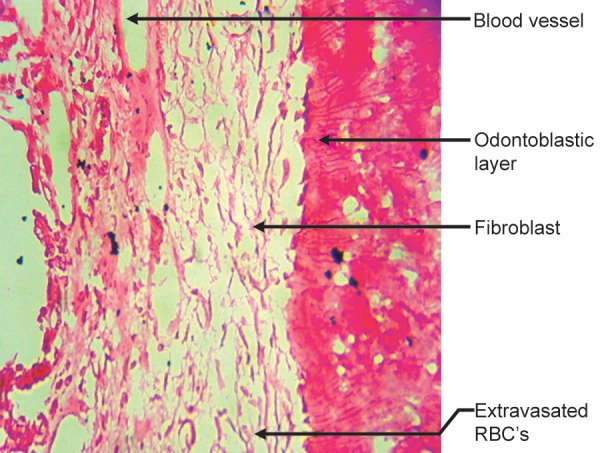
Root canal containing vital pulp tissue (H & E staining 40X)

Cross-section of root canal under 40X magnification shows vital pulp tissue containing blood vessels, odonto-blastic layer, fibroblast, extravasated RBS’s (Fig. 10).

## DISCUSSION

Aloe-Vera is native to Africa known by names lily of desert, plant of immortality or medicine plant.^4^ It is a very well grown plant in Rajasthan.

Plant is 99.5% water and remaining is active ingredients including essential oils, amino-acids, minerals, enzymes, and glycoprotein’s.^5^

### Chemical Composition of Aloe-Vera^6,7^

1. Antraquinones
  Aloin                        Ester of Cinnamic acid
  Barbaloin                  Aloe-emodin
  Isobarbaloin              Emodin
  Anthanol                   Chrysophanic acid
  Aloetic acid              Etheral oil
  Anthracine                Resistannol
2. Saccharides―Cellulose, Glucose, Mannose, L-rham-nose, Aldopentose, Acemannan.
3. Enzymes―Oxidase, Amylase, Catalase, Lipase, Alkaline Phosphatase.
4. Vitamins―B_1;_ B_2_, B_6_, Choline, Folic acid C, Alpha tocopherol, β carotene.
5. Inorganic―Ca, Na, Cl, Mn, Mg, Zn, Cu, Cr, Potassium sorbate.
6. Essential amino acid―Lysine, Threonine, Valine, Me-thionine, Leucine, Isoleucine, Phenylalanine.
7. Nonessential amino acids―Histidine, Arginine, Hydroxyproline, Aspartic acid, Glutamic acid, Proline, Glycerine, Alanine, Thyrosine.
8. Miscelaneous―Cholesterol, Triglycerides, Steroids, α sitosterol, Lignins, Steroids, Uric acid, Gibberel-lin, Lectin like substances, Salicylic acid, Mannose-6-phosphate.

Extensive research since 1930’s has shown that the clear gel has the dramatic ability to heal wounds, ulcer and burns by putting a protective coating on the affected areas and speeding up the healing rate.^8^

### Properties, Actions and Uses of Aloe-Vera Gel^9^

- Anti-inflammatory property
- Antibacterial property
- Antifungal property
- Antiviral property
- Moisturizing property
- Wound healing property
- Pain relief property
- Treatment of minor burns, skin abrasion and irriations
- Treatment of psoriasis and frostbite.

Anti-inflammatory Property

It was explained by Davis et al^10^ (1989), Thompson^11^ (1991) and Davis RH^12^ (1994). Davis RH,^13^ Hanley et al^14^ (1982) reported that Aloe-Vera extract (5% leaf homogenate) decrease inflammation by 48% in a rat adjuvant-induced arthritic inflammatory model.

Ito S et al^15^ (1993) reported that peptidase bradykinin isolated from aloe, breakdown bradykinin, an inflammatory substance that induces pain.

Three mechanisms explaining anti-inflammatory property are:^5^

1. Fujita and Teradaira^16^ (1976) said that carboxy-peptidase’s in aloe inactivate bradykinen which is a principle participant of inflammation.
2. Robson MC, Haggres WJ^17^ (1982) said that salicylates are by-products of amodin,aloe-emodin and aloin.
3. Klein AD^18^ (1980) said that magnesium lactate inhibits histidine decarboxylase, thereby preventing the formation of histamine from histidine in mast cells.

Keeping this property in mind, we did a study to prove anti-inflammatory action of gel in an endodontic procedure, pulpotomy.

Moisturizing Properties, Antibacterial, Antifungal and Antiviral Properties^19^

Bacteria inhibited by Aloe-Vera gel are *Streptococcus pyo-genus* and *Streptococcus faecalis,^20^ Pseudomonas aeruginosa,^21,22^* bacteria contributing to inflammation.

These properties were explained by Meadows TP^9^ (1980). He also used it for treatment of minor burns, skin abrasion, psoriasis and fross bite and for pain relief.

Accemannon reduced Herpes Simplex infection.

Hayes SM^23^ (1999) used it for lichen planus with systemic involvement.

C Choonahakarn et al^24^ (2007) said that Aloe-Vera gel is statistically more effective than placebo in inducing clinical and symptomatological improvement of oral lichen planus.

Wound Healing Property

High molecular weight polypeptide constituent from the gel demonstrated a healing effect on excisional wound in rats.

Yagi et al^25^ reported that Aloe-Vera gel contain a gly-coprotein with cell proliferation promoting activity. Then Davis et al noted that the gel improved wound healing by increasing blood supply, which increased oxygenation as a result.

In Thompson 1991 reported that topical application of the Aloe-Vera derivative allantoin gel stimulated fibroblast activity and collagen proliferation.

Mannose-6-phosphate component of the gel has been credited with a wound healing effect. Fibroblast proliferation was also observed *in vitro* and *in vivo* following treatment with carrisyn.

Shelton et al^26^ (1991) proved presence of salicylates in the gel giving aspirin like effects.

Haggers et al^27^ suggested that Aloe-Vera gel *in vitro* enhances wound healing process and inhibits growth of

Candida Albicans.

Garnick et al^28^ (1994) reported that Acemannon hydrogel in Aloe-Vera accelerates the healing of aphthous ulcers. Poor MR, Hall JE, Poor JS^29^ (2002) reported its healing effects on extraction sites.

Miscellaneous

Tellco CG, Ford P, Iocopino AM^30^ (1998) used the sticky and viscous nature, high bond strength and minimal toxicity of acemannon for denture adhesives.

Coronal pulp samples show presence of inflammatory cells like neutrophils (Fig. 7).

Studies suggest that the gel has an inhibitory action on arachidonic acid pathway via cyclooxygenase .Thus we can conclude that Aloe-Vera gel has potential antibacterial activity and thus it provides a scientific basis for the utilization of this plant in treatment of inflammatory process.

Based on promising results of the study, further studies can be done for its usage as an anti-inflammatory agent in endodontic procedure like pulpotomy, as it is cheap and affordable for common man.

## CONCLUSION

Aloe-Vera is of particular interest because it has found considerable popular acceptance as a home medication in western society, as well as being used in the traditional ethnic medicine of less developed countries.

There is evidence from scientific investigations reported in reputed journals that Aloe-Vera gel is of value at-least for burns and certain other dermatological conditions, and it does not have definite physiological effects. The “scientific”evidence for its rejection is almost countered by the “scientific” evidence for its beneficial properties.
